# Polymorphism Analysis of *Ch1* and *Ch2* Genes in the Siberian Cat

**DOI:** 10.3390/vetsci4040063

**Published:** 2017-12-01

**Authors:** Stefano Sartore, Eleonora Landoni, Sandra Maione, Alberto Tarducci, Antonio Borrelli, Dominga Soglia, Roberto Rasero, Paola Sacchi

**Affiliations:** Department of Veterinary Science, University of Turin, 10095 Grugliasco, Italy; eleonora.landoni@gmail.com (E.L.); sandra.maione@unito.it (S.M.); alberto.tarducci@unito.it (A.T.); antonio.borrelli@unito.it (A.B.); dominga.soglia@unito.it (D.S.); roberto.rasero@unito.it (R.R.); paola.sacchi@unito.it (P.S.)

**Keywords:** Fel d 1, Siberian cat, *Ch1* gene, *Ch2* gene, genetic selection, low-allergenic cat

## Abstract

Cats are usually spreaders of allergens that are critical for sensitive people; the Siberian cat is a breed supposed to be low level allergenic, according to some breeders’ statements. The sequence of the two genes, namely *Ch1* and *Ch2*, that code for the allergen Fel d 1, the major allergen responsible for outbreaks of allergy symptoms, is not yet known in the Siberian cat, and finding this was the aim of our investigation. Notably, our work is the first survey of the genetic structure of these genes in Siberian cats. The comparison of the sequences of Siberian cats, non-Siberian cats, and sequences present in the National Center for Biotechnology Information database revealed a considerable number of mutations; some of those detected in the Siberian cat, due to their position in exon regions, could affect the Fel d 1 allergenic properties. Therefore, further investigations are recommended to assess if the identified mutations can be responsible for a reduced-allergen synthesis and can be used as markers for selection of low level allergenic cats.

## 1. Introduction

Cats (*Felis catus*) are quite diffused domestic animals, admired for their beauty and their wayward temperament; unfortunately, they are also spreaders of allergens that are critical for allergic people, and in some extreme and dramatic cases the owners are sadly forced to remove the cat from their house to avoid health problems. These allergens have the potential to elicit the outbreak of symptoms with different degrees of severity: they can range from ordinary discomforts like rhinitis and conjunctivitis to potentially life-threatening asthmatic crisis [1,2,3]. In this regard, the selection of low allergenic cats could be a new possibility for allergic people who wish to keep in their home their favorite pet. Fel d 1, the major allergen produced by cats, is a tetrameric protein of 35–40 kDa, constituted of two heterodimer of 18–20 kDa [4,5,6]. Each subunit comprises two polypeptide chains, chain 1 and chain 2, encoded by two separated genes, namely *Ch1* and *Ch2*. Fel d 1 is a secretoglobulin primarily produced in sebaceous, anal, and salivary glands and is present on the animal skin and pelt as a consequence of the grooming [7,8,9]. Although allergic patients react with a wide number of proteins found in cat pelts [10,11], Fel d 1 is the most prominent allergen, and the clinical accuracy of this finding was confirmed by investigations that detected the Fel d 1-specific IgE in the serum of about 80% of cat-allergic patients [10,11,12]. It is worth noticing that houses with cats have often airborne levels of Fel d 1, which is more than enough to provoke an asthma crisis in highly sensitive inhabitants [13,14]. In a previous survey [15], the existence of hypoallergenic cats due to a lower production of Fel d 1 protein was stated; thus, the aim of this investigation was to analyze the sequences of the two genes (*Ch1* and *Ch2*) encoding Fel d 1 protein in some Siberian cats, a supposed low level-allergenic breed accordingly to some breeders statement. Notably, our work is the first survey of the genetic structure of these genes in Siberian cats to identify molecular differences between Siberian and non-Siberian cats.

## 2. Materials and Methods

Blood samples were collected from 39 cats, namely 4 Siberian and 35 non-Siberian. Three Siberian cats were previously tested for the Fel d 1 concentration in saliva (private lab, Charlottesville, VA, USA), resulting in 2.19, 1.66, and 0.48 µg/mL. DNA was extracted with NucleoSpin Blood Quick Pure Kit (Macherey-Nagel GmbH, Duren, Germany), and *Ch1* and *Ch2* genes were amplified by PCR with a 2720 AB Thermal Cycler Device following a protocol suggested by the literature [16]. The primers sequences were as follows:

- 5′GGGGATCCTGGAACACCATGTTAGACGCAG3′ (Forward)
- 5′GGGAATTCTGCAGTTACCTTTAACACAGAG3′ (Reverse) for *Ch1*;
- 5′GGGCTGCAGATTCTAGTCAGCCTGATTGA3′ (Forward)
- 5′GGGGATCCTGACACCATGAGGGGGGCA3′ (Reverse) for *Ch2*.

The amplicons from four Siberian and five non-Siberian cats were analysed by the NGS (Next Generation Sequencing) technique with the use of the Miseq system (Illumina, CA, USA). The Variant Calling for each sample was performed using the Miseq Reporter software tool (Illumina, CA, USA). Mutation points in Siberian and Short-Haired cats amplicons were compared with National Center for Biotechnology Information (NCBI, [17]) sequences X62477 (*Felis catus* gene for Fel d 1 protein chain 1) and X62478 (*Felis catus* gene for Fel d 1 protein chain 2). The graphic representation of the mutation points identified was realized in NCBI Genome Workbench tool.

## 3. Results

The amplifications provided fragments of approximately 1700 bp and 2500 bp from *Ch1* and *Ch2*, respectively.

**Figure 1:**
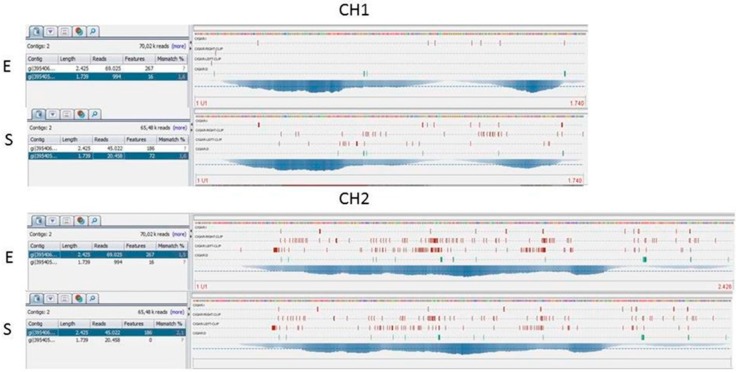
Graphic representation of the sequencing coverage and alignment of the reference sequence of the *Ch1* and *Ch2* genes. E = Short Haired cat. S = Siberian cat.

For *Ch1*, sequencing data from one Siberian and one non-Siberian were discarded because they provided a very low quantity of reads. More than 90% of both genes were covered with an average depth exceeding 200X. On average, 88.5% of *Ch1* (from about base 150 to base 1700) had a sequence suitable for variant calling (read depth >20), except for a gap between base 1094 and base 1194, probably due to a repetitive region. For *Ch2*, 84.2% of genes (from about base 212 to base 2250) had a read depth >20X (Figure 1).

The comparison between Short-Haired cats and NCBI X62477(DNA)/NM_001048153.1(mRNA) and X62478(DNA)/NM_001048154.1(mRNA) revealed 36 mutations in *Ch1* and 88 mutations in *Ch2*, whereas Siberian cats compared with the same reference sequences revealed 45 mutations in *Ch1* and 93 mutations in *Ch2*. Focusing the attention on mutation points identified in Siberian cats compared with Short-Haired cats, 13 mutations in *Ch1* and 8 mutations in *Ch2* were detected in Siberian only, namely 11 mutations in *Ch1* intron 3 and 7 in *Ch2* introns 1 and 2. In exon 1 of *Ch1*, one Siberian cat showed a silent mutation in position 119 on X62477 (g.119T > C). In the same region of the gene, another Siberian cat showed a missense mutation causing a p.A13S substitution in position 138 (g.138G > T) (Table 1, No. 37); in *Ch2*, the same cat showed a missense mutation causing a p.N85S substitution in position 2231 on X62478 (g.2231A > G). In all the Siberian cats, a deletion of 6 nucleotides causing the loss of 2 Serines (p.S85_S86del) was localized, corresponding to c.260_265delAGCTCC on NM_001048154.1; the deletion was also present on X62478 starting from position 2230, and it was shared with some but not all the Short-Haired cats.

In 2 Siberian cats, the concentration of Fel d 1 in saliva was >1.5 µg/mL, whereas it decreased below 0.5 µg/mL in the subject showing the missense mutations (Table 1, No. 37).

## 4. Discussion

Cats are spreaders of allergens and the consequences of this characteristic for allergic people can be serious; in the cases of rhinitis and conjunctivitis, the cohabitation arrangement needs to be adjusted, e.g., by ventilating and cleaning rooms and clothes properly and constantly, while having asthma clearly makes it impossible to own a cat [18,19]. Despite the small number of samples, namely 4, and given the fact that subjects of this breed are very rare primarily because of the scarcity of breeding farms and their high cost, in Siberian cats we detected a considerable number of mutations in *Ch1* and *Ch2*, the two genes encoding for Fel d 1 allergen. Some of these mutations, due to their position in exon regions, may play a key role in the allergenic properties of the Fel d 1 protein.

In *Ch1*, one Siberian cat shows a missense mutation; namely, in the signal sequence an Alanine hydrophobic residue is replaced by a polar one of Serine that could modify the internalization into endoplasmic reticulum and modify the glycosylation pattern. In *Ch2*, the same Siberian cat shows one mutation in exon 3, namely a missense mutation that replaces a potential *N*-glycosylation site of Asparagine with a new *O*-glycosylation site of Serine. If the absence of 2 Serine residues in close proximity is also taken into account, these mutations could change the structure of the mature glycoprotein and thus its allergenic properties; the low concentration of Fel d 1 in the saliva of this subject could be related to the low production of the intact allergenic molecule. The number of Siberian cats showing this molecular variant must be well established, as well as the level of Fel d 1 in Siberian cats with different variants. 

The remaining mutations are positioned in introns and their role is still a matter of investigation.

## 5. Conclusions 

Further analyses are recommended to assess if the identified mutations are responsible for a reduced allergen synthesis and then if they can be used as markers for selection of low-level allergenic cats. In the case of positive confirmation, the relevance of this finding would consist in also giving sensitive people the possibility of going beyond their allergy and finally owning a cat in their home.

## Figures and Tables

**Table 1:** Main mutations affecting the coding sequence of *Ch1* and *Ch2* genes in some Siberian cats: position on a GenBank reference sequence, effects on amino acid sequence, and Fel d 1 concentration in saliva. Minus and plus signs mean absence or presence of the mutation.

Cat No.	Mutation *Ch1* X62477	Effect	Mutation *Ch2* NM_001048154.1	Effect	Mutation *Ch2* X62478	Effect	Fel d 1 (µg/mL)
	g.138G > T	p.A13S	c.260_265delAGCTCC	p.S85_S86del	g.2231A > G	p.N85S	
21	−		+		−		unknown
37	+		+		+		0.48
38	−		+		-		2.19
39	Not amplified		+		−		1.66
